# Irisin—A Pancreatic Islet Hormone

**DOI:** 10.3390/biomedicines10020258

**Published:** 2022-01-25

**Authors:** Daniel Norman, Carl Johan Drott, Per-Ola Carlsson, Daniel Espes

**Affiliations:** 1 Department of Medical Cell Biology, Uppsala University, 751 23 Uppsala, Sweden; Daniel.norman@mcb.uu.se (D.N.); carljohan.drott@mcb.uu.se (C.J.D.); per-ola.carlsson@mcb.uu.se (P.-O.C.); 2 Department of Medical Sciences, Uppsala University, 751 85 Uppsala, Sweden; 3 Science for Life Laboratory, Department of Medical Cell Biology, Uppsala University, 751 23 Uppsala, Sweden; 4 Science for Life Laboratory, Department of Medical Sciences, Uppsala University, 751 85 Uppsala, Sweden

**Keywords:** irisin, pancreatic islets, blood flow, vascular

## Abstract

Irisin is a myokine involved in glucose homeostasis. It is primarily expressed in skeletal muscle, but also in the pancreas. This study aimed to elucidate its presence and role in the islets of Langerhans—i.e., its effect on insulin and glucagon secretion as well as on blood flow in the pancreas. The precursor of irisin, fibronectin type III domain-containing protein 5 (FNDC5), was identified in rat and human islets by both qPCR and immunohistochemistry. Both α- and β-cells stained positive for FNDC5. In human islets, we found that irisin was secreted in a glucose-dependent manner. Neither irisin nor an irisin-neutralizing antibody affected insulin or glucagon secretion from human or rat islets in vitro. The insulin and glucagon content in islets was not altered by irisin. The intravenous infusion of irisin in *Sprague Dawley* rats resulted in nearly 50% reduction in islet blood flow compared to the control. We conclude that irisin is an islet hormone that has a novel role in pancreatic islet physiology, exerting local vascular effects by diminishing islet blood flow without affecting insulin secretion per se.

## 1. Introduction

Irisin is a myokine related to exercise that was first discovered as a secreted peptide in mouse skeletal muscle in 2012. It is cleaved from its precursor fibronectin type III domain-containing protein 5 (FNDC5) [1]. Irisin is known to increase the expression of mitochondrial uncoupling protein 1, converting white adipose tissue (WAT) into brown-like adipose tissue [2,3]. The net effect is weight reduction and improved glucose metabolism [1,4]. Therefore, irisin holds the potential to reverse obesity. Induced modest weight reduction as well as lowered insulin and glucose levels in obese high-fat diet mice have, consistent with this, been reported previously [1]. 

Skeletal muscle is considered as the main source of FNDC5 based on its high expression and the fact that plasma irisin levels correlate positively with muscle mass [5,6]. However, FNDC5 is also found in tissues involved in energy homeostasis, such as adipose tissue and the liver, although with a 100–200 times lower expression than skeletal muscle [5,6,7,8]. Despite many reported effects, the receptor for irisin still remains unknown, except in osteo- and adipocytes, where the involvement of the receptor integrin αV/β5 has been suggested [9]. In the pancreas, a low mRNA expression of FNDC5 has been observed, without regard to cell type, [6] and in a histology-based study, the islets of Langerhans stained positive for FNDC5/irisin, indicating that this could be an islet hormone [10]. Interestingly, irisin has been found to enhance glucose-stimulated insulin secretion (GSIS) in human and murine islets as well as in INS-1E cells [11]. In another study, irisin improved glucose tolerance while reducing serum insulin levels in a rat model of type 2 diabetes mellitus (T2D) [12]. Lower levels of irisin have been observed in gestational diabetes and T2D [13,14,15], while we and others have reported that irisin levels are higher in individuals with type 1 diabetes mellitus (T1D) [16,17]. 

Irisin relaxes mesenteric arteries in mice via the endothelial nitric oxide-guanosine 3′ 5′ cyclic phosphate-dependent pathway, but also through mechanisms independent of the endothelium [18]. Moreover, irisin improves endothelial function in the aorta of high-fat diet mice through the AMP-activated protein kinase and endothelial nitric oxide synthase pathways [19]. Another study demonstrated that irisin protects against vascular endothelial injury and atherosclerosis through the same pathways in diabetic mice [20]. 

Despite the seemingly intimate relationship between irisin and glucose metabolism, the role of irisin in the endocrine pancreas has not been thoroughly investigated. Since irisin has effects that could be beneficial in T2D, investigating its pancreatic role could lead to novel treatments. The aim of this study is, therefore, to evaluate the presence, expression and secretion of irisin in pancreatic islets and, in addition, its effect on insulin secretion and pancreatic blood flow.

## 2. Materials and Methods

### 2.1. Animals

Male *Sprague Dawley* rats (Taconic, Ry, Denmark) 12–16 weeks of age and weighing approximately 300–400 g and *C57 BL/6 mice* (M&B, Ry, Denmark) 10–12 weeks of age and weighing 25–30 g were housed with free access to pellets and water. All animal protocols and experiments were approved by the Animal Ethics Committee for Uppsala University, Uppsala, Sweden (C65/16, 2016-07-01). The guide for the care and use of laboratory animals, eighth edition [21] was followed, as well as specific national laws, where applicable.

### 2.2. Human Tissue

The ethical board in Uppsala approved the use of human pancreatic tissue for this study (2017/283, 2017-08-09). Human islets were obtained from the Nordic Network for Clinical Islet Transplantation (Rudbeck Laboratory, Uppsala University Hospital, Uppsala, Sweden). 

### 2.3. Islet Isolation and Culture

Human islets were cultured in CMRL1066 medium (Cellgro/Mediatech, Manassas, VA, USA) at a glucose concentration of 5.6 mmol/L, with the addition of 10% (vol/vol) bovine serum, L-glutamine (2 mmol/L; Sigma-Aldrich, St Louis, MO, USA) and benzylpenicillin (100 U/mL; Roche Diagnostics, Bromma, Sweden). Rat islets were isolated by the collagenase digestion of the pancreas, as previously described [22].

### 2.4. Immunohistochemistry

Pancreatic sections from human donors, *Sprague Dawley* rats and *C57BL/6* mice were stained for FNDC5, insulin, glucagon and nuclei. Staining was carried out by deparaffinizing the sections, followed by heating in a cooker. For blocking, 3% donkey serum/PBS was used for 30 min, followed by overnight incubation at 4 °C with primary antibodies for FNDC5 (Rabbit polyclonal antibody bs-8486R, Bioss, Woburn, MA, USA) and insulin (Cat No. 20-IP30, Fitzgerald, Acton, MA, USA) or glucagon (Biotin conjugated antibody, Cat No. 13-9743-82, eBioscience™, ThermoFisher scientific, Waltham, MA, USA). For secondary antibodies, Alexa 488 conjugated donkey anti-rabbit (Cat. No. 711-545-152, Jackson ImmunoResearch Laboratories, Ely, United Kingdom), Alexa 594 conjugated donkey anti-guinea pig (Cat. No. 706-585-148, Jackson ImmunoResearch Laboratories, Ely, United Kingdom) and Cy3 conjugated Streptavidin (Cat. No. 016-160-084, Jackson ImmunoResearch Laboratories, Ely, United Kingdom) were used and incubated for 1 h. Nuclei were stained by applying a DAPI solution for 5 min. Fluorescent immunohistochemistry images were acquired using a Zeiss LSM 780 (Zeiss, Jena, Germany) confocal and evaluated by comparing the overlap of FNDC5 with insulin and glucagon separately.

### 2.5. FNDC5 Expression

To investigate the relative expression of *FNDC5* in islets compared to skeletal muscle, total RNA from islets isolated from human donors (*n* = 6) and rats (*n* = 3) was extracted according to the manufacturer’s instructions (RNeasy Plus Micro Kit, Qiagen AB, Kista, Sweden). Purity was determined using a NanoDrop 2000C spectrophotometer (Thermo Scientific, Waltham, MA, USA). All RNA samples had an OD 260/280 between 1.9 and 2.2. Human skeletal muscle total RNA was purchased from Ambion (Invitrogen, Life Technologies, Stockholm, Sweden) and rat skeletal muscle total RNA was purchased from Takara Bio (636220, Saint-Germain-en-Laye, France). For total RNA from islets and skeletal muscle in human and rat, qPCR for the genes *FNDC5*, *glyceraldehyde 3-phosphate dehydrogenase (GAPDH)* and *Ribosomal Protein S7 (RPS7)* was performed using a Light Cycler 480 (Roche Diagnostic, Mannheim, Germany) and LightCycler FastStart DNA Master PLUS SYBR Green I kit (Roche Diagnostic, Mannheim, Germany). See the Supplementary Material for the primer details (Table S1). Primer specificity was confirmed by a melting curve analysis with a single peak and agarose gel electrophoresis with a single band at the anticipated size. The relative expression of *FNDC5* was calculated using the housekeeping genes *RPS7* and *GAPDH* separately; the calculation was performed by averaging the Ct-values from duplicates followed by the ΔΔ-Ct method.

### 2.6. Islet Perifusion

To evaluate irisin secretion from the islets, groups of 50 size-matched human islets (*n* = 7 donors) were inserted into filter-covered perifusion chambers (Suprafusion 1000, 6 channel system, Brandel, Gaithersburg, MD, USA). The islets were perifused (200 µL/min) with Krebs ringer bicarbonate buffer (KRBH) supplemented with 2 mg/mL of bovine serum albumin with low (3.33 mmol/L) or high (33.3 mmol/L) glucose concentrations. The islets were first perifused with low glucose for 30 min to acquire a baseline secretion. Perifusion was then performed with low glucose for 12 min followed by high glucose for 37 min, then again with low glucose for 14 min. In addition, the same protocol was performed but with the potentiation of insulin release by adding forskolin (1 μmol/L) to the KRBH. Released irisin was analyzed using an ELISA (#EK-067-29, Phoenix Peptides Europe GmbH, Karlsruhe, Germany). 

### 2.7. Irisin Incubation and Insulin Release

After isolation and 48–72 h of incubation, islets were moved to 6-well plates, with each well containing 2 mL of RPMI 1640 cell medium (Sigma-Aldrich, R0883, St Louis, MO, USA) and 100 islets. RPMI 1640 cell media had been supplemented with 10% vol/vol fetal bovine serum prior to the experiments (Sigma-Aldrich, F7524, St Louis, MO, USA), 1% vol/vol L-glutamine (Sigma-Aldrich, G7513, St Louis, MO, USA) and 0.2% vol/vol penicillin/Streptomycin (50,000 U/mL and 50 mg/mL, respectively; 11074440001, Sigma-Aldrich, St Louis, MO, USA). Irisin (100 nmol/L, recombinant Irisin, 067-29A, Phoenix Peptides Europe GmbH, Karlsruhe, Germany) was added to the treatment groups (Table 1) and islets were incubated for another 24 h before insulin release experiments.

Five groups were incubated with or without 100 nmol/L irisin for 24 h or during release (Table 1). One group had no irisin but an irisin-neutralizing antibody was added (Recombinant Anti-FNDC5 antibody, ab174833, Abcam, Cambridge, United Kingdom).

After incubation, GSIS was performed with triplicates of ten islets in each group. Islets were exposed to a 1.67 mmol/L glucose solution consisting of KRBH with 2 mg/mL bovine albumin for one hour at 37 °C. The medium was saved and replaced with an identical solution but with 16.7 mmol/L glucose added and incubated for another hour. Then, the medium was saved and all islets were sonicated in distilled water and added to 95% acid ethanol. Insulin and glucagon concentrations in release medium and sonicated islets were determined by ELISA (human or rat insulin and glucagon ELISA, respectively, Mercodia, Uppsala, Sweden).

### 2.8. Blood Flow Measurements

To investigate the effect of irisin on pancreatic blood flow, measurements of blood flow were performed with a microsphere technique, as previously described in detail [23,24,25] and briefly as follows. 

Black, non-radioactive microspheres (1–2 × 10^6^, E-Z Trac; IMT, Irvine, CA, USA) were injected into *Sprague Dawley* rats after the infusion of irisin dissolved in saline (6.25 µg/mL, 2.0 mL/h; recombinant Irisin, 067-29A, Phoenix Peptides Europe GmbH, Karlsruhe, Germany) or saline alone (2.0 mL/h) for 60 min, via the right carotid artery into the ascending aorta. An arterial blood reference sample was collected for 60 s from the catheter in the femoral artery starting immediately before the injection of microspheres. The exact flow in the femoral artery was confirmed in each experiment by weighing the sample. The pancreas, adrenal glands, duodenum, colon, kidneys and skeletal muscle were retrieved, dissected free from adipose tissue and weighed. In addition, white and brown adipose tissue was dissected free and weighed. All tissue samples were subjected to a freeze–thawing technique to visualize the microspheres and allow the separate counting of intra-islet microspheres [23]. The microspheres present in the samples were counted in a microscope equipped with both dark- and bright-field illumination [23]. The reference blood collected during the experiment was transferred to glass microfiber filters with a pore size of <10 µm before the microspheres therein were counted. The organ blood flow was then calculated according to the formula $Q_{org}=N_{org}\times Q_{ref}÷N_{ref}$, where $Q_{org}$ denotes organ blood flow (mL/min), $Q_{ref}$ denotes the flow of the reference sample (mL/min), $N_{org}$ denotes the number of microspheres present in the organ and $N_{ref}$ denotes the number of microspheres present in each reference sample. In the adrenal glands, a difference in blood perfusion between the left and right glands of less than 15% was used as the measurement for the equal distribution of the microspheres; otherwise, the experiment was discarded. 

The dose of irisin used in this study was chosen based on previous experimental in vivo studies and clinical studies of circulating levels of irisin [16,26].

### 2.9. Statistical Analysis

All values are expressed as mean ± SEM. For insulin release data, the mean of triplicates of islets in each incubation from each animal or human donor was considered as one observation. Parametric data with only two groups were analyzed with Student’s two-tailed t-test for unpaired and paired observations. To compare multiple groups with parametric paired data, analysis of variance (ANOVA) with Geisser–Greenhouse correction was used, together with multiple comparisons using Tukey’s post hoc test. For all comparisons, a *p*-value < 0.05 was considered statistically significant. Calculations were performed using the statistical software Prism 8 (GraphPad Software, San Diego, CA, USA).

## 3. Results

### 3.1. Immunohistochemistry

**Figure 1:**
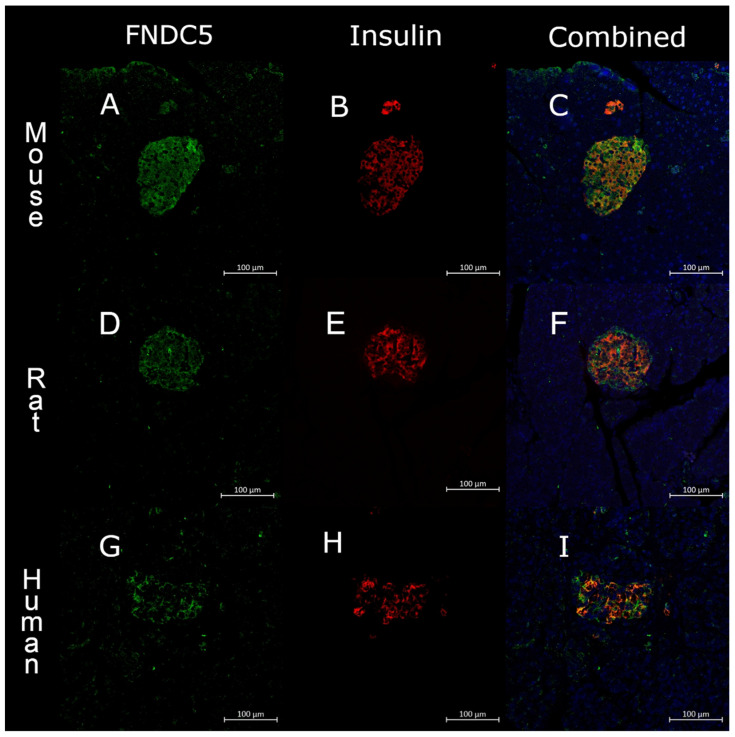
Confocal image of a single islet of Langerhans from (**A**–**C**) mouse, (**D**–**F**) rat and (**G**–**I**) human split into fibronectin type III domain-containing protein 5 (FNDC5, green), insulin (red) and a combined picture. Scale bar = 100 μm.

**Figure 2:**
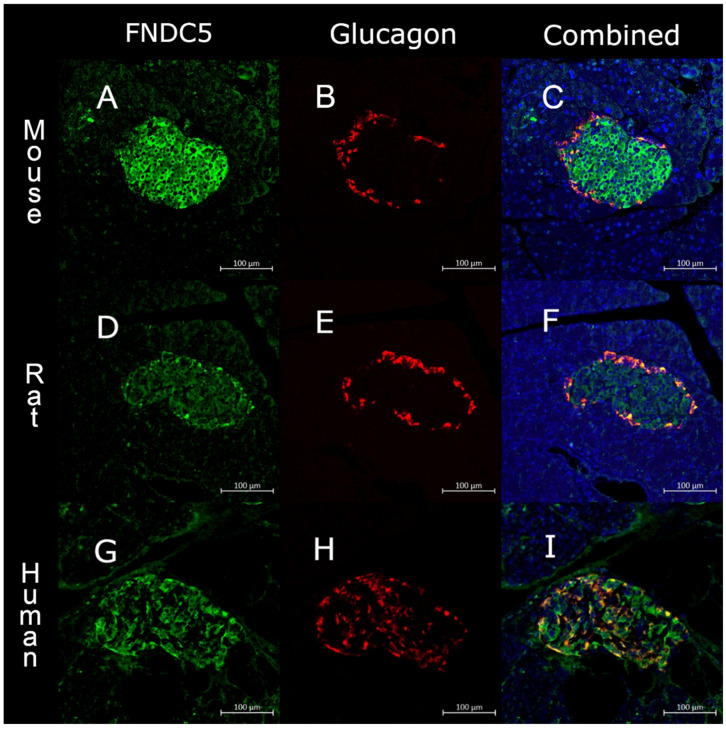
Confocal image of a single islet of Langerhans from (**A**–**C**) mouse, (**D**–**F**) rat and (**G**–**I**) human split into FNDC5 (green), glucagon (red) and a combined picture. Scale bar = 100 μm.

Pancreatic islets in mouse, rat and human showed a whole islet staining for FNDC5, colocalizing with both insulin and glucagon (Figure 1 and Figure 2). The specificity of the antibody was confirmed by blocking the antibody with irisin peptide (Figure S1). 

### 3.2. Gene Expression

Both human and rat pancreatic islets were found to express *FNDC5*. The relative expression compared to skeletal muscle was 1.1–48.5% in human and 0.6–8.9% in rat (Table 2). 

### 3.3. Islet Perifusion

**Figure 3:**
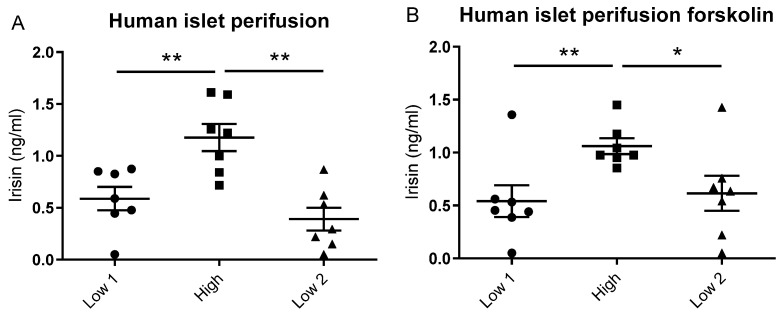
Mean irisin concentration in the effusate at the sequential perifusion of human islets with a low (3.33 mmol/L), a high (33.3 mmol/L) and a second low glucose concentration in (**A**) human islets (*n* = 7) without forskolin and (**B**) human islets (*n* = 7) with forskolin (1.0 μmol/L) at both the low and high glucose concentrations. All values are given as means ± standard error of the mean (SEM). * denotes *p* < 0.05 and ** *p* < 0.01.

In human islets, the secretion of irisin increased in response to glucose during in vitro islet perifusion experiments (Figure 3A). The potentiation of insulin release by forskolin did not alter the secretion of irisin during either low- or high-glucose conditions (Figure 3B).

### 3.4. Insulin Release In Vitro

**Figure 4:**
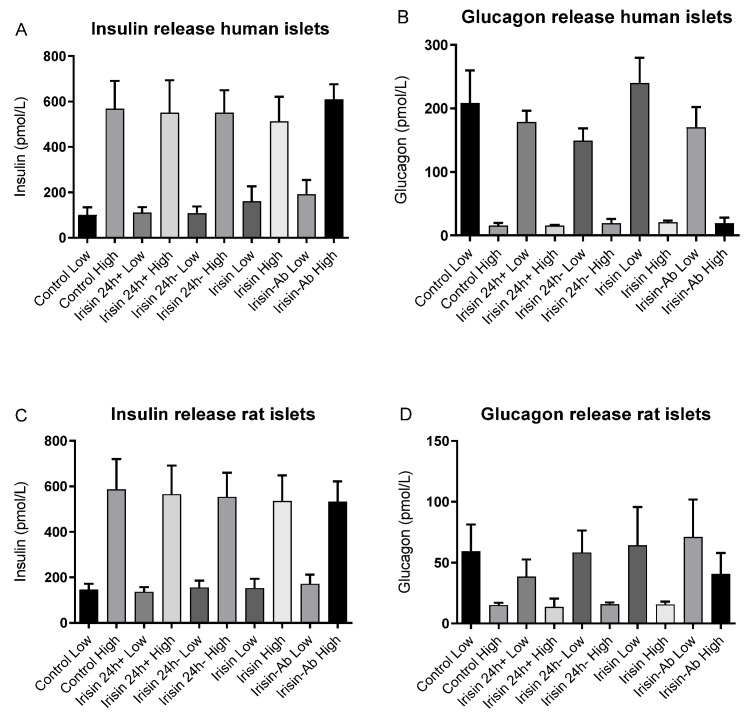
Concentrations of insulin and glucagon in the medium after incubation with low (1.67 mmol/L) and high (16.7 mmol/L) glucose concentrations in (**A**,**B**) human islets (*n* = 5) and (**C**,**D**) rat islets (*n* = 6) in the control groups, irisin in both preincubation and incubation (24+), irisin in preincubation (24 h−), and irisin in incubation media and anti-irisin antibody, as further specified in Table 1. All irisin incubations were 100 nmol/L. All values are given as means ± SEM.

**Figure 5:**
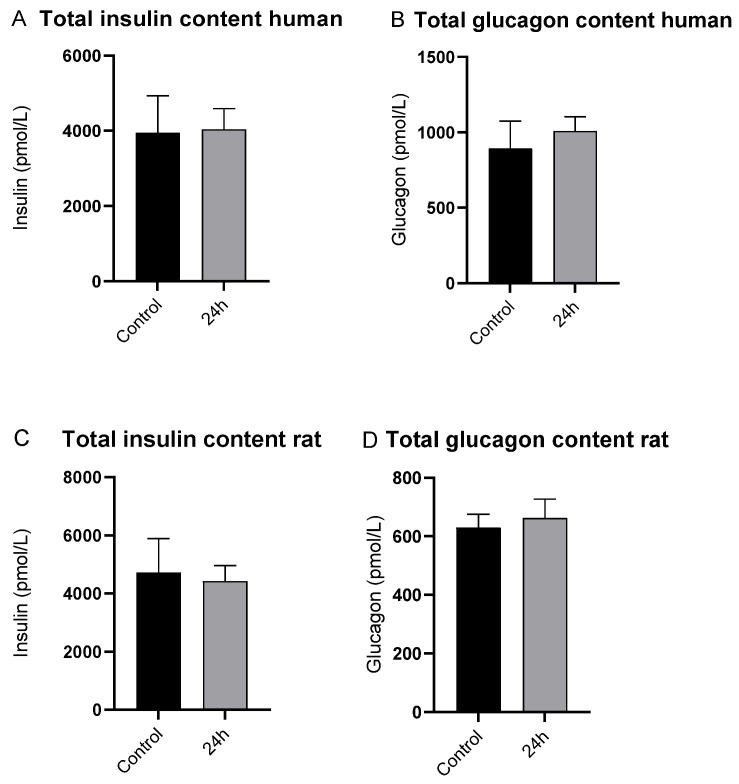
Total content of insulin and glucagon in islets after incubation and glucose-stimulated insulin secretion, compared between control and the two groups incubated with irisin (100 nmol/L) for 24 h in (**A**,**B**) human islets (*n* = 5) and (**C**,**D**) rat islets (*n* = 5). All values are given as means ± SEM.

Neither irisin nor an irisin-neutralizing antibody affected insulin or glucagon secretion from human and rat islets during low and high glucose incubations (Figure 4A–D). There was no difference in the total content of insulin or glucagon in the islets after 24 h irisin incubation compared to the control (Figure 5A–D).

### 3.5. Blood Flow Measurements

**Figure 6:**
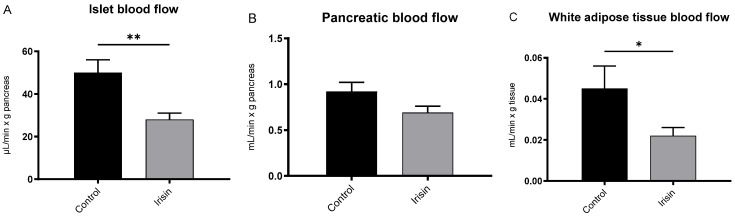
Blood flow in rats infused for 60 min with either saline as control (black) or irisin (grey, 100 nmol/L) for (**A**) islet blood flow, (**B**) pancreatic blood flow (*p* = 0.074, unpaired *t*-test) and (**C**) white adipose tissue. All values are given as means ± SEM for 6 and 8 rats, in control and irisin groups, respectively. * denotes *p* < 0.05 and ** denotes *p* < 0.01 by Student’s unpaired *t*-test.

All rats maintained a mean arterial blood pressure of 100–120 mmHg during the in vivo experiments which was not affected by the administration of saline or irisin. Pancreatic islet blood flow (IBF) was reduced by nearly 50% compared to the control after the infusion of irisin (Figure 6A). There was also a tendency towards a decreased whole pancreatic blood flow (*p* = 0.07; Figure 6B). In addition, irisin decreased the blood flow in WAT by nearly 50% compared to the control (Figure 6C). However, irisin did not affect the blood flow of brown adipose tissue (BAT), or any of the other examined tissues (duodenum, colon, kidney and skeletal muscle).

## 4. Discussion

Previous histological studies of the pancreas have only demonstrated that FNDC5/irisin is present in the exocrine and endocrine pancreas, but not in which cell types [10]. Our histological result replicates this expression pattern but also suggests that in the islets, α- and β-cells, and possibly even more cell-types in mouse, rat and human islets, contain FNDC5. The expression of *FNDC5* was also confirmed in both human and rat islets by qPCR, further specifying the previously known expression in whole pancreatic samples [6]. In addition, the secretion of irisin from human pancreatic islets during perifusion experiments further strengthens the notion that irisin is an islet hormone.

Interesting similarities exist between irisin and glucagon-like peptide 1 (GLP-1), the latter of which is used in the treatment of type 2 diabetes as a GLP-1 receptor agonist. They share common features, such as an increased secretion in response to carbohydrates and lipids [27], and seem to be interconnected in the regulation of glucose metabolism, since both irisin synthesis and GLP-1 secretion are increased by metformin treatment [28,29]. In addition, 12 weeks of treatment with a GLP-1 analog increased the levels of irisin in T2D patients [30]. Specifically in the pancreas, both have previously been shown to increase β-cell survival, proliferation and insulin secretion [27]. In addition, similar to what we observed for irisin, GLP-1 has been found to reduce IBF [31]. These similarities raise interest in the potential pharmaceutical effect of irisin in type 2 diabetes. However, the increase in GSIS by irisin proposed to be shared with GLP-1 could not be replicated in our study.

Incubating rat or human islets with irisin had no effect on the secretion of insulin or glucagon at either low or high glucose concentrations. Neither was the insulin nor glucagon content increased after 24 h of irisin incubation. This contradicts a previous study where GSIS and insulin content was increased after irisin incubation in human and murine islets as well as in rat INS-1E cells [11]. However, multiple methodological differences are present. For GSIS, the previous study used a 1.5-times higher stimulatory glucose level for murine islets and INS-1E cells and only three separate experiments were carried out for murine and human islets. For total insulin content, only three experiments were used in the previous study and the islets were lysed in lysis buffer, whereas we sonicated them. In our study, this was performed after the GSIS experiment, while in the study by Natalicchio et al. this was conducted as a separate experiment. In addition, for both GSIS and total insulin content, they normalized data for total protein content while we did not normalize them, in accordance with the only comparison regarding the normalization of GSIS data we have found so far [32]. Considering that other studies have found that irisin improves GSIS under glucolipotoxic conditions [12,33] and that irisin is antiapoptotic for INS-1 cells [11,12,33], it could be that the increased GSIS is an indirect effect through antiapoptotic effects—i.e., more cells survive during the preincubation steps and hence secrete more insulin. Regardless of the effect being direct or indirect, irisin is still interesting pharmaceutically, since it could potentially inhibit the loss of β-cell mass in gluco- and lipotoxic conditions, which is common in T2D.

The notion that irisin may play a role in glucotoxic conditions is strengthened by our finding that irisin secretion from human islets is positively correlated to glucose levels. Although this secretion is unlikely to affect plasma levels, this finding potentially demonstrates glucose to be a regulating factor in the secretion of irisin, in addition to exercise. 

It was noteworthy that irisin selectively reduced pancreatic IBF but not PBF. This vascular effect is in line with previous results showing irisin to have a role in endothelial function and the regulation of vasculature [18,19,20]. A lowering of IBF by irisin in exercise, during which irisin levels have been seen to increase [34], would be fitting, since insulin secretion is inhibited during moderate exercise by sympathetic innervation and circulating catecholamines [35], thereby requiring a lower blood flow. Considering that irisin is proliferative and anti-apoptotic for β-cells, the decreased blood flow could also be an additional protective mechanism for islets during glucotoxic conditions.

Taking together its similarities with GLP-1 and its potential role in protecting the islets, irisin could be a new pharmaceutical candidate in T2D. Considering the global increase in the incidence of T2D and the effects it has on both individual patients and society, new and better treatments are needed.

In summary, irisin is a hormone present in—and secreted by—pancreatic islets, exerting local effects on both the endocrine cells and vasculature, and can therefore be considered an islet hormone. Irisin is potentially of importance for the treatment of diabetes due to its seemingly protective effects on pancreatic islets during high-glucose conditions. This confirms that the receptor for irisin deserves mechanistic studies that could lead towards a new potential treatment for T2D.

## Figures and Tables

**Table 1:** The five groups with different treatments preceding glucose-stimulated insulin release (GSIS).

Group	Treatment
Control	Control
Irisin 24 h+	24 h incubation with 100 nmol/L irisin and irisin in release media
Irisin 24 h−	24 h incubation with 100 nmol/L irisin
Irisin	Regular incubation and 100 nmol/L irisin in release media
Irisin Ab	Regular incubation and 100 nmol/L irisin antibody in release media

**Table 2:** Relative expression of the gene *fibronectin type III domain-containing protein 5 (FNDC5)* in rat and human islets compared to skeletal muscle, calculated using the housekeeping genes *glyceraldehyde 3-phosphate dehydrogenase (GAPDH)* and *Ribosomal Protein S7 (RPS7)* separately. Each value represents one animal or human donor.

	Rat—*GAPDH* (%)	Rat—*RPS7* (%)	Human—*GAPDH* (%)	Human—*RPS7* (%)
	5	1	17	7
	9	1	12	11
	8	1	19	8
			48	6
			5	1
			6	1
Mean	7.3	1	17.8	5.7

## Data Availability

Data will be made available upon reasonable request.

## Supplementary Materials

The following are available online at https://www.mdpi.com/article/10.3390/biomedicines10020258/s1, Figure S1: (A,B) Skeletal muscle from C57 BL/6 mice stained for FNDC5 and DAPI without recombinant irisin in (A) and with recombinant irisin added to the antibody mix to extinguish the signal in (B); (C,D) Rat islets stained for FNDC5 (green), insulin (red) and DAPI in combined pictures without recombinant irisin in (C) and with recombinant irisin in the antibody mix in (D), Table S1: Sequences and references for primers used.
