# Supplementary material for: Irisin—A Pancreatic Islet Hormone
Source: Biomedicines. 2022 Jan 25;10(2):258. doi: 10.3390/biomedicines10020258 (PMC8869325; doi:10.3390/biomedicines10020258)
Supplement: Supplementary file 1 [file biomedicines-10-00258-s001.zip › biomedicines-1505449-SI.pdf]

Table S1. Sequences and references for primers used.

Gene	Orientation	Primer sequence or commercial primer
FNDC5 Human	F	GGCCTCCAAGAACAAAGATGAG
	R	GCAGAAGAGGGCAATGACAC
FNDC5 Rat	F	TCTTCATGTGGGCAGGTGTCAT
	R	GGTGCTGGTCTCTGATGCACT
GAPDH Human	F	GTCAAGGCTGAGAACGGGAA
	R	AAATGAGCCCCAGCCTTCTC
GAPDH Rat	Commercial primer	PrimePCR™ Template for SYBR® Green Assay: Gapdh, Rat (BioRad, Hercules, California, USA)
RPS7 Human	F	GAAGTTGGTGGTGGTCGGAA
	R	TGCACAGCTGTCAGAGTACG
RPS7 rat	Commercial primer	PrimePCR™ Template for SYBR® Green Assay: Rps7, Rat (BioRad, Hercules, California, USA)

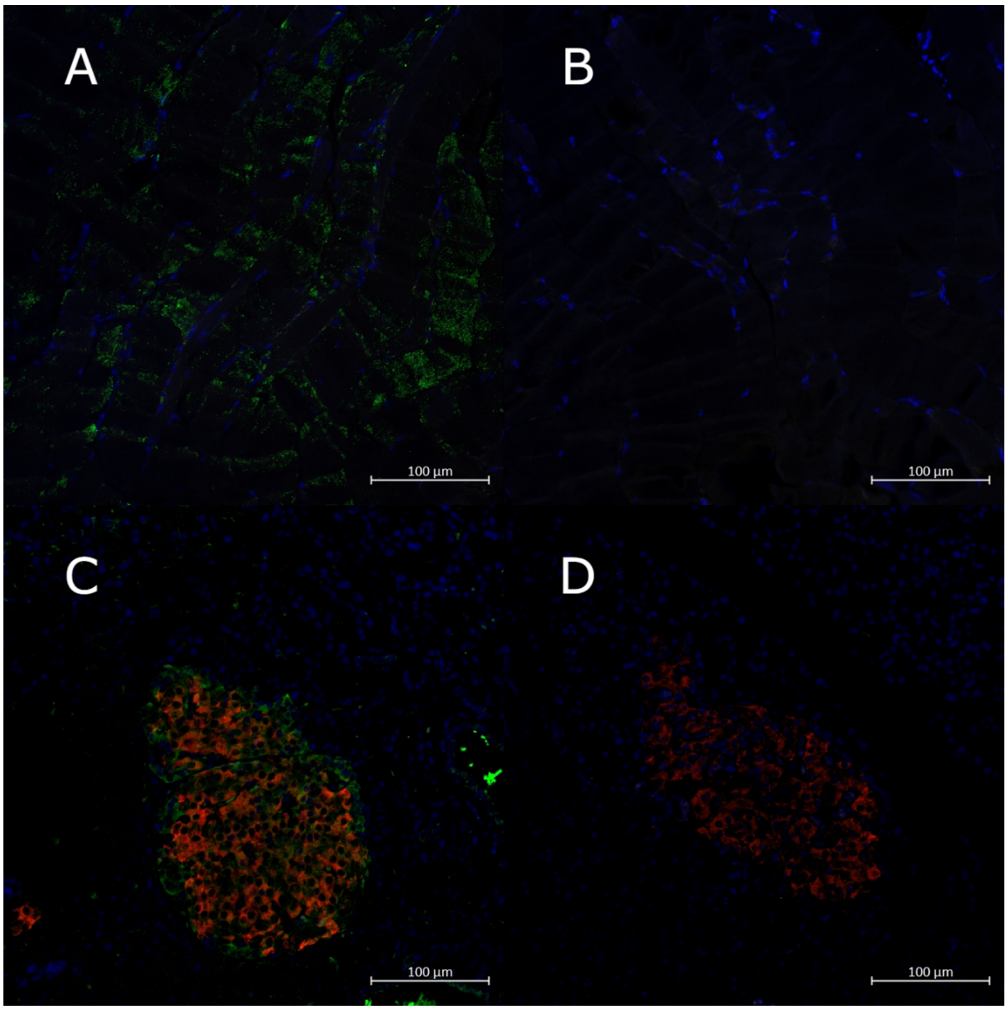

Figure S1. (A–B) Skeletal muscle from C57 BL/6 mice stained for FNDC5 and DAPI without recombinant irisin in **(A)** and with recombinant irisin added to the antibody mix to extinguish the signal in **(B); (C–D)** Rat islets stained for FNDC5

(green), insulin (red) and DAPI in combined pictures without recombinant irisin in **(C)** and with recombinant irisin in the antibody mix in **(D)**.
